# Comparison of the influence of three fibroid treatment options: supracervical hysterectomy, ulipristal acetate and uterine artery embolization on ovarian reserve – an observational study

**DOI:** 10.1186/s13048-018-0420-1

**Published:** 2018-06-01

**Authors:** Piotr Czuczwar, Anna Stepniak, Pawel Milart, Tomasz Paszkowski, Slawomir Wozniak

**Affiliations:** 3rd Department of Gynecology of the Medical University of Lublin, ul. Jaczewskiego 8, 20-954 Lublin, Poland

## Abstract

**Background:**

To assess and compare the influence of three fibroid treatment options: supracervical hysterectomy, ulipristal acetate and uterine artery embolization on ovarian reserve.

**Methods:**

Prospective, observational, open-label study performed at the 3rd Chair and Department of Gynecology of the Medical University of Lublin, Poland. Premenopausal Caucasian women with symptomatic uterine fibroids were recruited into 3 groupspatients qualified for supracervical hysterectomies; patients qualified for preoperative ulipristal acetate (UPA) treatment scheduled for supracervical hysterectomies or myomectomies; patients qualified for uterine artery embolization (UAE). The following markers of ovarian reserve were investigated: antral follicle count (AFC), anti-Mullerian hormone (AMH), inhibin B (INHB), follicle stimulating hormone (FSH) and estradiol (E2). These markers were assessed before and 3 months after supracervical hysterectomies, before and 3 months after UAEs, and before and after 3 months of UPA treatment, before the scheduled surgeries. Baseline characteristics (age, parity, dominant fibroid volume, hemoglobin level, BMI, as well as AFC, AMH, INHB, FSH and E2) were compared between the study groups by Kruskall-Wallis ANOVA. Pre- and post-interventional values of AFC, AMH, INHB, FSH and E2 in the studied groups were compared with the Wilcoxon matched pairs test.

**Results:**

Twenty-six, 27 and 30 patients were included in the final analysis in the supracervical hysterectomy, UPA and UAE groups, respectively. Three months after supracervical hysterectomy INHB and E2 significantly decreased, while AFC, AMH and FSH remained unchanged. After 3 months of UPA treatment the values of all the assessed markers of ovarian reserve were not significantly different in comparison to baseline. Conversely, three months after UAE the values of AFC, AMH, INHB, and E2 were significantly decreased, while FSH was significantly increased.

**Conclusions:**

Of the compared fibroid treatment methods UAE seems to have the greatest impact on ovarian function and should not be offered to patients concerned about their ovarian function. Supracervical hysterectomy did not affect the most accurate markers of ovarian reserve, and therefore appears to be safe in terms of ovarian function. UPA did not change any of the studied markers of ovarian reserve and seems a reasonable option when ovarian function is concerned.

## Background

Surgical management constitutes the majority of fibroid treatment, but minimally-invasive alternatives are also available [1]. Uterine artery embolization (UAE) is a procedure, during which embolic material is administered into the uterine arteries to decrease the blood supply of fibroids [2]. It has previously been shown, that UAE results in reduction of fibroid symptoms and improvement of the quality of life [2]. Recent studies have demonstrated the efficacy of ulipristal acetate (UPA), a selective progesterone receptor modulator (SPRM), in the medical management of fibroids [3].

Many women seek alternative treatment options for fibroids because of their concern about future fertility, and, interestingly, irrespective of the desire for childbearing [4]. Premature menopause may have significant negative impact on the future quality of life and morbidity, such as increased risk of osteoporosis, cardiovascular disease, and all-cause mortality [5].

The impact of various fibroid treatment options on ovarian function has been debated [6]. The influence of fibroid surgery and UAE on ovarian reserve has previously been investigated in numerous studies but the possible decline of ovarian reserve after these interventions remains controversial [7]. Available data on the effects of SPRMs on ovarian reserve are scarce [8, 9]. The aim of this study was to investigate the influence of three fibroid treatment methods (supracervical hysterectomy, UAE and UPA) on ovarian reserve.

## Methods

This prospective observational open-label single center study was performed at the 3rd Chair and Department of Gynecology of the Medical University of Lublin, Poland. Patients were recruited from December 2012 to November 2013 and followed-up for 3 months. Three groups of patients with symptomatic fibroids were recruited: patients qualified for supracervical hysterectomies with bilateral salpingectomy; patients qualified for preoperative UPA treatment before supracervical hysterectomies or myomectomies; patients qualified for UAE. Due to the capacity of biochemical kits and the expected patient numbers the number of patients was restricted to 29, 29 and 30 in the respective groups. Because UPA patients were scheduled to undergo surgeries 3 months after the beginning of UPA treatment, follow-up was limited to 3 months in all groups. It must be emphasized, that even though patients receiving UPA underwent surgeries after the treatment, the assessment of ovarian reserve in the UPA group was performed before the surgical interventions, because the objective of this study was to assess the possible impact of UPA on ovarian reserve, and not of both UPA and surgical treatment. Inclusion criteria were: symptomatic uterine fibroids, abnormal uterine bleeding, dominant fibroid diameter of 30–80 mm, intramural location of fibroids, normal ovarian reserve (anti-Mullerian hormone (AMH) (> 1.0 ng/ml) and follicle stimulating hormone (FSH) (< 20 mIU/ml) on days 2–4 of the menstrual cycle and regular menstrual bleeding), no history of ovarian pathology, normal endometrial biopsy, normal endometrial appearance on ultrasound (according to current guidelines [10]) and no previous hormonal treatment. Baseline characteristics (age, parity, dominant fibroid volume, hemoglobin level, BMI) were recorded in all patients. Fibroid volume was estimated by transvaginal ultrasound using the ellipsoid formula.

In patients receiving UPA therapy (Esmya©, Gedeon Richter, Budapest, Hungary), 5 mg/24 h of UPA was administered orally during the first 4 days of menstrual bleeding and continued for 3 months. Indications for UPA therapy included a planned myomectomy/hysterectomy because of abnormal uterine bleeding, anemia and pelvic pain. Patients were qualified for UAE according to the Clinical Practice Guidelines of the Society of Obstetricians and Gynaecologists of Canada [11, 12]. During UAE polyvinyl alcohol particles were administered until complete stasis of contrast had been achieved in both uterine arteries. Only patients with successful bilateral embolization of the uterine arteries were included in the study. Written informed consent was obtained from all participants and the study protocol was accepted by the local bioethics committee. This study was performed according to the STROBE Statement Checklist for observational studies.

The following markers of ovarian reserve were investigated: antral follicle count (AFC), AMH, inhibin B (INHB), FSH and E2. AFC was measured according to current guidelines [13] between the 2–4 day of the cycle using a Samsung Medison V20 Prestige (Samsung Medison, Seoul, South Korea) equipped with a transvaginal probe. All antral follicles of 2–10 mm in diameter were included. AFC > 10 was considered to be normal. To determine the time for follow-up assessment after supracervical hysterectomies, the patients were asked to perform ovulation tests and/or present for ultrasound monitoring of the cycle.

FSH, E2, AMH and INHB levels were measured in plasma samples collected between the 2–4 day of the cycle by commercially available Enzyme-linked Immunosorbent Assay Kits according to the manufacturer’s instructions. The following kits were used: E90228Hu for AMH (Uscn Life Science Inc., Wuhan, Hubei, China); E90760Hu for INHB (Uscn Life Science Inc., Wuhan, Hubei, China); FR E-2400 for FSH and FR E-2000 for E2 (Labor Diagnostika Nord GmBH & Co. KG, Nordhorn, Germany). The following cut-points for normal values were used: < 20 mIU/ml for FSH; > 50 pg/ml for E2; > 1 ng/ml for AMH; and > 10 pg/ml for INHB.

### Statistics

Statistical analysis was performed using Statistica 12 (Statsoft, Tulsa, OK, USA). The normality of continuous variables was tested with the Shapiro–Wilk test. Baseline characteristics (age, parity, dominant fibroid volume, hemoglobin level, BMI, as well as AFC, AMH, INHB, FSH and E2) were compared between the study groups by Kruskal-Wallis ANOVA. Pre- and post-interventional values of AFC, AMH, INHB, FSH and E2 in the studied groups were compared with the Wilcoxon matched pairs test. To confront the impact of the studied treatment options on AFC, AMH, INHB, FSH and E2, percentage changes of these parameters were calculated for each study group and compared by Kruskal-Wallis ANOVA with post-hoc analysis. Percentages of cases with abnormal post-interventional values of AFC, AMH, INHB, FSH and E2 were compared between the studied groups with the Chi square test. *P* values < 0.05 were considered significant.

## Results

### Participants

**Fig. 1:**
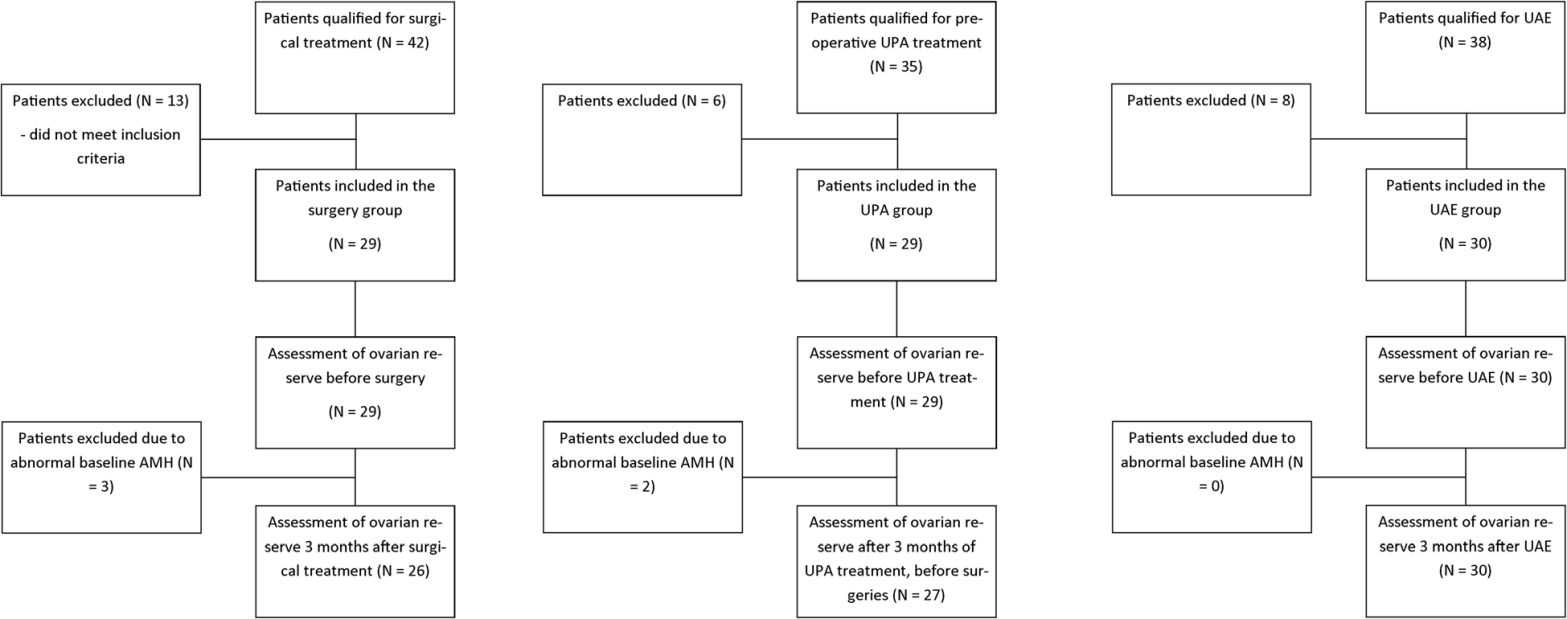
Flow-chart of the study design

Flow-chart of the study design is shown on Fig. 1. Forty-two patients qualified for supracervical hysterectomies with bilateral salpingectomies, 35 qualified for preoperative UPA treatment and 38 qualified for UAE were screened to obtain the required number of patients in the study groups (29, 29 and 30 respectively). After performing the biochemical tests at the end of the study 3 patients were excluded from the supracervical hysterectomy group and 2 from the UPA group due to abnormal baseline AMH levels, leaving 26, 27 and 30 patients in the study groups, respectively.

Patient demographic characteristics are shown in Table 1. Age, parity, dominant fibroid volume, hemoglobin levels and BMI were not significantly different between the studied groups. Similarly, baseline AFC values and plasma levels of AMH, INHB, FSH and E2 were not significantly different.

**Table 1:** Baseline characteristics of the patients qualified for hysterectomy, ulipristal acetate treatment or uterine artery embolization

	Supracervical hysterectomy	UPA	UAE	*P*
Number of patients	26	27	30	N/A
Age	38 (36–43)	37 (34–43)	35 (33–40)	> 0.05
Parity	2 (1–2)	2 (1–2)	1 (1–2)	> 0.05
Dominant fibroid Volume (cm^3^)	103.3 (87.6–127.6)	104.6 (85.2–132.1)	107.75 (87.4–131.1)	> 0.05
Hemoglobin (g/dl)	10.5 (9.0–11.9)	10.2 (8.9–11.1)	10.3 (9.1–11.4)	> 0.05
BMI (kg/m^2^)	27.5 (23.6–31.3)	28.1 (24.1–32.9)	26.9 (22.5–30.6)	> 0.05

Data are presented as median (interquartile range). *N/A* not applicable, *UPA* ulipristal acetate, *UAE* uterine artery embolization

### Main results

Pre- and post-interventional AFC values and plasma levels of AMH, INHB, FSH and E2 are shown in Table 2. Three months after supracervical hysterectomy INHB and E2 significantly decreased, while AFC, AMH and FSH remained unchanged. After 3 months of UPA treatment the values of all the assessed markers of ovarian reserve were not significantly different in comparison to baseline. Conversely, three months after UAE the values of AFC, AMH, INHB, and E2 were significantly decreased, while FSH was significantly increased. Percentage changes of the investigated markers of ovarian reserve are shown in Table 3. In all studied markers of ovarian reserve percentage changes at 3-month follow-up were significantly greater after UAE than after supracervical hysterectomy or UPA. There were no significant differences in percentage changes of ovarian reserve markers between the supracervical hysterectomy and UPA groups (Table 3). The percentage of cases with abnormal post-interventional values at 3-month follow-up in the supracervical hysterectomy, UPA and UAE groups was 19.2; 18.5 and 56.7% for AFC (*p* = 0.006), 3.9; 3.7 and 36.7% for AMH (*p* <  0.001), 15.4; 18.5 and 46.7% for INHB (*p* = 0.014), 11.5; 18.5 and 43.4% for FSH (*p* = 0.015) and 0; 0 and 43.3% for E2 (*p* <  0.001), respectively.

**Table 2:** Parameters assessed before and 3 months after hysterectomy, ulipristal acetate therapy or uterine artery embolization

	Baseline	3 months follow-up	*P*
AFC Hysterectomy	14 (12–24)	14 (9–19)	> 0.05
AFC UPA	15 (13–24)	15 (12–19)	> 0.05
AFC UAE	19 (15–24)	8 (4–12)	< 0.001
AMH Hysterectomy	2.64 (2.03–3.84)	2.66 (1.91–3.67)	> 0.05
AMH UPA	2.89 (2.11–4.34)	2.91 (2.22–4.12)	> 0.05
AMH UAE	3.04 (2.53–3.7)	1.28 (0.49–2.11)	< 0.001
INHB Hysterectomy	28.09 (20.76–35.33)	24.05 (18.79–32.74)	< 0.001
INHB UPA	25.16 (14.78–47.34)	26.78 (13.78–42.33)	> 0.05
INHB UAE	25.11 (15.67–33.9)	10.78 (4.71–15.83)	< 0.001
FSH Hysterectomy	8.32 (5.51–13.47)	9.6 (5.62–12.59)	> 0.05
FSH UPA	11.21 (4.56–16.78)	9.88 (6.12–19.34)	> 0.05
FSH UAE	8.75 (6.3–11.12)	16.99 (12.35–41.33)	< 0.001
E2 Hysterectomy	81.85 (73.62–108.12)	74.59 (59.34–115.43)	0.005
E2 UPA	90.23 (78.34–136.45)	94.32 (67.22–154.33)	> 0.05
E2 UAE	98.44 (87.32–115.89)	57.37 (39.34–93.22)	< 0.001

Data are presented as median (interquartile range). *AFC* antral follicle count, *AMH* anti-Mullerian hormone, ng/ml, *INHB* inhibin B, pg/ml, *FSH* follicle stimulating hormone, mIU/ml, *E2* estradiol, pg/ml, *UPA* ulipristal acetate, UAE uterine artery embolization

**Table 3:** Median percentage changes in the investigated markers of ovarian reserve 3 months after supracervical hysterectomy, uterine artery embolization or after a course of UPA therapy

Variable	Study group	Percentage change at 3-month follow-up	*P*	Post-hoc analysis
AFC	Hysterectomy	−8.4 (− 15 – − 4.2)	< 0.001	Hysterectomy vs UPA: NS
	UPA	−7.8 (−14.3–0)		Hysterectomy vs UAE: *p* < 0.001
	UAE	− 63.4 (− 78.9 – − 37.5)		UPA vs UAE: *p* < 0.001
AMH	Hysterectomy	−3.5 (− 10–4.8)	< 0.001	Hysterectomy vs UPA: NS
	UPA	−5.8 (− 7.7 – − 5.1)		Hysterectomy vs UAE: *p* < 0.001
	UAE	− 61.9 (− 82 – − 47.4)		UPA vs UAE: p < 0.001
INH	Hysterectomy	−7.4 (−15.1 – − 2.5)	< 0.001	Hysterectomy vs UPA: NS
	UPA	− 7.0 (− 10.6–3.0)		Hysterectomy vs UAE: *p* < 0.001
	UAE	−60.7 (− 74.5 – − 51.2)		UPA vs UAE: p < 0.001
FSH	Hysterectomy	21.5 (−9.1–35)	< 0.001	Hysterectomy vs UPA: NS
	UPA	12.7 (− 9.8–27)		Hysterectomy vs UAE: *p* < 0.001
	UAE	125.6 (51–295.8)		UPA vs UAE: p < 0.001
E2	Hysterectomy	−13.3 (− 19.5 – − 3.5)	< 0.001	Hysterectomy vs UPA: NS
	UPA	−3.3 (− 10.3–11.4)		Hysterectomy vs UAE: *p* < 0.001
	UAE	−41 (−53.5–7.6)		UPA vs UAE: p < 0.001

Data are presented as median (interquartile range). *AFC* antral follicle count, *AMH* anti-Mullerian hormone, *INHB* inhibin B, *FSH* follicle stimulating hormone, *E2* estradiol, UPA ulipristal acetate, *UAE* uterine artery embolization

At 3-month follow-up, abnormal uterine bleeding had ceased in all cases in the UPA and UAE groups and no significant side-effects were observed. In the surgery group all patients underwent supracervical hysterectomies with bilateral salpingectomies, both ovaries were spared in all cases, no complications in the post-operative course were noted.

## Discussion

The main finding of this study is that UAE had the greatest impact on ovarian reserve. Supracervical hysterectomy affected only INHB and E2 levels, while no significant changes in AFC, AMH and FSH were observed. UPA did not affect any of the investigated markers of ovarian reserve. Percentage changes of the investigated parameters were significantly greater in the UAE group than in the UPA and hysterectomy groups. Similarly, the percentage of patients with abnormal post-interventional ovarian reserve markers was the greatest in the UAE group.

Ovarian reserve is understood as the number and quality of remaining oocytes and describes individual reproductive potential. There is no uniformly accepted definition of decreased ovarian reserve [14]. The following tests are used to assess ovarian reserve: FSH, E2, INHB, AMH and the clomiphene citrate challenge test as well as AFC and ovarian volume. AMH is accepted to be the most accurate test [14].

The possible mechanism of compromising ovarian reserve seems to be the clearest for hysterectomies. Three hypotheses are considered [6]. Transecting the utero-ovarian ligament with the ovarian branch of the uterine artery decreases the blood supply of the ovary and may lead to ischemia and loss of primordial follicles [15]. The underlying indication for the hysterectomy, such as fibroids or abnormal uterine bleeding, may also have a significant impact on ovarian reserve [16]. Finally, it has been speculated that the uterus might secrete paracrine factors regulating the recruitment of follicles, the loss of which could possibly diminish ovarian reserve [6]. For UAE it seems most likely that the embolic material may reach the ovary and block the ovarian microvasculature resulting in minor ischemia and reduction of the primordial follicular pool [6]. Similarly as in the case of hysterectomies, the presence of fibroids may be responsible for the lower ovarian reserve in general [6]. For UPA, this subject has not been debated.

The influence of UAE on ovarian reserve has been previously investigated but remains controversial. In a randomized trial by Mara et al. comparing the outcomes of UAE and myomectomy in fibroid patients, 6 months after the procedure significantly more patients after UAE had elevated FSH levels [17]. The randomized EMMY trial compared FSH and AMH levels after hysterectomy and UAE in premenopausal women with symptomatic fibroids within a 2-year follow-up. The risk of ovarian failure (defined as an increase in FSH > 40 mIU/ml) was similar in both the hysterectomy and UAE groups. However, only in the UAE group a significant decrease in AMH levels was observed throughout the study period [18]. In a recently published update of the EMMY trial the authors extended the follow-up to 10 years and reported, that in both groups a similar percentage of patients underwent menopause [19]. The authors concluded that both hysterectomy and UAE affect ovarian reserve. Conversely, Tropeano et al. did not observe significant changes in FSH, AFC and ovarian volume for 5 years after UAE [20]. This difference might be because the women in the EMMY trial were on average 10 years older than in the Tropeano et al. study and younger age may have a protective effect on ovarian function after UAE [6]. Some authors suggested that the impact of UAE on the hormonal markers of ovarian reserve may be transient. Tsikouras et al. reported a significant increase of FSH after UAE, that reached its maximum value at 3 months and returned to baseline values 12 months after the procedure [21]. The authors also investigated changes in AMH levels and reported significant decreases 1 month after UAE in younger patients (≤ 45 years) and 1 and 3 months after UAE in older patients (> 45 years). Kim et al. showed a significant decrease of AMH and AFC at 3 and 12 months after UAE [22]. AMH levels remained low after 12 months of follow-up compared to the expected AMH levels. Interestingly, in patients < 40 years-old a significant recovery of AMH between the 3 and 12 months measurements was observed, again suggesting that the ovaries in younger women may be more likely to recover after UAE [22]. Basing on our results and the literature data it seems that there is at least a short-term effect of UAE on ovarian reserve and this treatment option should not be offered to patients concerned about their ovarian function.

Surprisingly, we did not observe significant changes in AMH, AFC and FSH after supracervical hysterectomies. Two large prospective cohort studies investigating the association between hysterectomy and menopause were published: the Farquhar et al. and the PROOF study [16, 23]. Both studies concluded, that hysterectomy is associated with an earlier onset of menopause. However, both studies used only FSH as a marker of ovarian function and the occurrence of menopause as an endpoint. Moreover, it was not clear whether the surgical intervention or the indication for surgery contributed to the earlier onset of menopause [6]. Nevertheless, this appears to be in contradiction with our results. However, in the abovementioned studies many patients underwent unilateral oophorectomies during the surgeries. Moreover, an update of the PROOF trial has been recently published [24]. The authors compared AMH levels in women undergoing hysterectomies with the control group 1-year after the surgeries. In general, patients undergoing hysterectomies had lower AMH levels compared with the control group. Surprisingly, these differences were not seen among white women, but remained significant in black women [24]. This is in concordance with the results of our study, where only white women were included. Interestingly, the EMMY trial, in which hysterectomy did not exert a permanent effect on AMH levels, also included mainly white women [18]. There is some data suggesting that reproductive function differs by race. It has been shown that AMH levels were significantly lower in black women than in white [25]. It may be speculated that white race is a protective factor against the negative effect of hysterectomy on ovarian reserve.

In our study UPA did not affect any of the markers of ovarian reserve. Two previous studies assessed ovarian function after UPA treatment. UPA (up to 10 mg daily) did not affect the levels of E2 and FSH and follicular growth on ultrasound after 84 days of treatment [8]. In a study assessing the long-term efficacy and safety of UPA (four 12-week courses) E2 levels remained stable [9]. Even though E2 and FSH are not reliable markers of ovarian reserve, these results support the statement, that UPA treatment does not affect ovarian reserve.

Most of novel treatment algorithms for fibroids begin with UPA treatment [26] and therefore the influence of this treatment on ovarian reserve could play an important role, especially in patients wishing to conceive. Taking into consideration that an increasing number of pregnancies after UPA treatment is being reported [27], our observation that UPA treatment does not affect the markers of ovarian reserve, further supports the role of UPA in the treatment of patients wishing to preserve fertility.

The strength of our study is that it is the first prospective trial comparing the impact of 3 different fibroid treatment options on ovarian function, in a homogenous population. Many factors were suggested to influence the outcome of fibroid treatment, especially in case of UAE. These include fibroid features such as volume, vascularity and location, but also patient characteristics such as age and menopausal status [28]. Even factors such as patient race may completely change the impact of hysterectomy on ovarian reserve [24]. Therefore, it is difficult to compare the outcomes of various fibroid treatment options observed in different populations. However, our results may not be generalized to other populations.

Our study has some limitations. The main limitation is the short follow-up. During the data collection for this study, UPA was registered only for a single 3-month course of preoperative treatment, which limited the follow-up duration. Recently, the safety of up to 8 consecutive 3-month UPA courses has been showed [29], which encourages to assess the impact of extended UPA treatment on ovarian function. It is difficult to state, whether the observed reduction of AMH levels 3 months after UAE is maintained over a longer time period. As previously discussed, some studies suggested that the decrease of AMH at 1–3 months after UAE is transient and recovers within a year, at least in younger patients [20–22]. Other studies showed that this phenomenon may be prolonged (up to 2 years follow-up), especially in older patients [18, 22]. Further studies are necessary to fully elucidate this issue and identify the possible factors influencing ovarian function after UAE. Our study is relatively small and open-label rather than randomized, therefore some differences in the baseline characteristics of the studied groups that could have affected the final results might not have been identified. However, most of the patients presenting for fibroid treatment had their treatment preference and we decided that it would be more ethical to take it into consideration and make shared decisions with the patients.

## Conclusions

In conclusion, UAE seems to have the greatest impact on ovarian function and should not be offered to patients concerned about their ovarian function. Supracervical hysterectomy did not affect the most accurate markers of ovarian reserve and appears to be safe in terms of ovarian function. UPA did not change any of the studied markers of ovarian reserve and seems a reasonable option when ovarian function is concerned.
